# The tibial growth plate as a predictor of the original tibial plateau joint line as a reference for kinematically aligned total knee arthroplasty

**DOI:** 10.1186/s13018-017-0708-7

**Published:** 2018-01-08

**Authors:** Katsuya Nagai, Yasuo Niki, Kengo Harato, Shu Kobayashi, Takeo Nagura, Takayuki Nakamura, Morio Matsumoto, Masaya Nakamura

**Affiliations:** 1 Department of Orthopaedic Surgery, School of Medicine, Keio University, 35 Shinanomachi, Shinjuku, Tokyo, 160-8582 Japan; 2 DePuySynthes Joint Reconstruction, 700 Orthopaedic Drive, Warsaw, IN 46582 USA

**Keywords:** Kinematically aligned total knee arthroplasty, Joint line, Growth plate, Tibial plateau

## Abstract

**Background:**

Restoration of the natural joint line is a cornerstone for kinematically aligned total knee arthroplasty (TKA). The purpose of this study was to investigate the relative orientation of the tibial growth plate (GP) with respect to the tibial plateau (TP) for possible application in predicting natural joint line for knees with highly advanced osteoarthritis patient at the time of kinematically aligned TKA.

**Methods:**

Images from computed tomography (CT) of 27 normal knees (9 males, 18 females; mean age, 31.6 years) were studied. Geometry of the GP was extracted from CT images, and its moment-of-inertia axes were calculated for the whole GP and the medial and lateral halves. Angular orientations of each GP axis with respect to the TP plane were measured in anatomical coordinates.

**Results:**

The TP and GP planes were oriented in 2.3 ± 1.8° of varus and 1.1 ± 1.9° of valgus relative to the tibial mechanical axis, respectively. With respect to the TP plane, the whole GP plane was inclined in 3.4 ± 1.5° of valgus. Orientation of the GP plane differed drastically between medial and lateral halves. The medial GP was in 4.9 ± 2.9° of varus and 1.8 ± 2.5° of anterior inclination, and the lateral half was in 10.4 ± 2.4° of valgus and 18.6 ± 4.0° of anterior inclination relative to the TP.

**Conclusions:**

Angular orientation of the original TP plane can be predicted in reference to the GP plane and may provide reasonable guidance for the target bone resection angle of the tibia in kinematically aligned TKA.

## Background

From the perspective of patient satisfaction after total knee arthroplasty (TKA), interest has been growing in surgical techniques that utilize modified implant alignments. Several investigators have demonstrated no significant correlation between neutral mechanical alignment and implant survivorship [1–3], and a slight under-correction of limb alignment yielded more satisfactory outcomes [4]. Kinematically aligned (KA)- or anatomically aligned (AA)-TKA techniques aim to reproduce a natural joint line and the flexion-extension axis of the femur in a pre-osteoarthritis (OA) state presumed in each patient. Functional recovery after KA-TKA has recently been reported to be better than that after mechanically aligned TKA [5, 6].

Restoration of the natural joint line is a cornerstone philosophy for KA- or AA-TKA, but reliable methods have yet to be described for predicting the original joint line at the time of preoperative planning of TKA. Pereira et al. [7] examined relationships between the joint line and six bony landmarks in the proximity of the joint line, concluding that two bony landmarks were at constant distances from the joint line. However, at least three landmarks are required to reproduce a three-dimensional (3D) joint line in the form of a plane. The proximal growth plate (GP) of the tibia can provide a potential base of reference to original articular surface of the tibial plateau (TP), since the GP is relatively easy to visualize in 3D from computed tomography (CT) images. The purpose of this study was to investigate the orientation of the tibial GP with respect to the TP surface and to establish a method of reproducing joint line orientation, for possible application in predicting the pre-OA natural joint line for KA- or AA-TKA.

## Methods

**Fig. 1:**
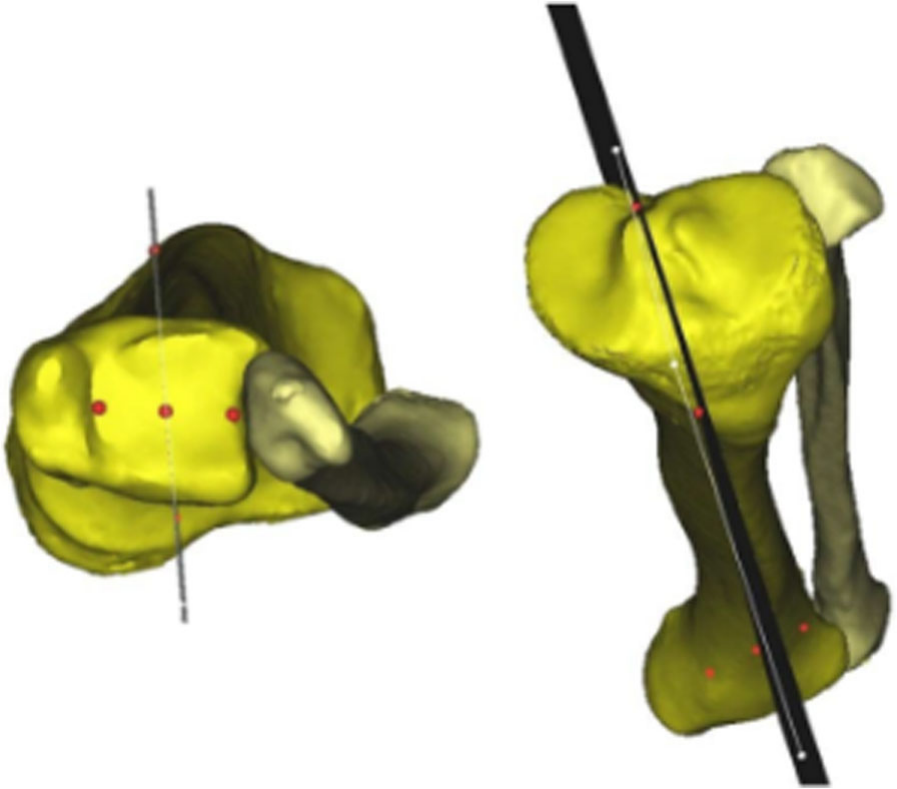
Definition of the tibial coordinate system and the mid-sagittal plane

Participants comprised 27 subjects (9 males, 18 females) with a mean age of 31.6 ± 13.8 years. All subjects underwent CT of the whole lower limb. Primary diagnoses of patients included anterior or posterior cruciate ligament rupture due to apparent sports injury. No patients presented with radiographic OA. All anthropometric measurements were made on 3D tibial models created from CT data using Mimics® (Materialise NV, Leuven, Belgium). To standardize the measurements, tibial models were aligned to a previously reported anatomical landmark-based coordinate system [8]. In this method, the mid-sagittal plane of the tibia was defined by three anatomical landmarks: the medial margin of the tibial tuberosity, the center of the posterior cruciate ligament (PCL) origin, and the apex of the tibial plafond as the center of the foot joint (Fig. 1).

**Fig. 2:**
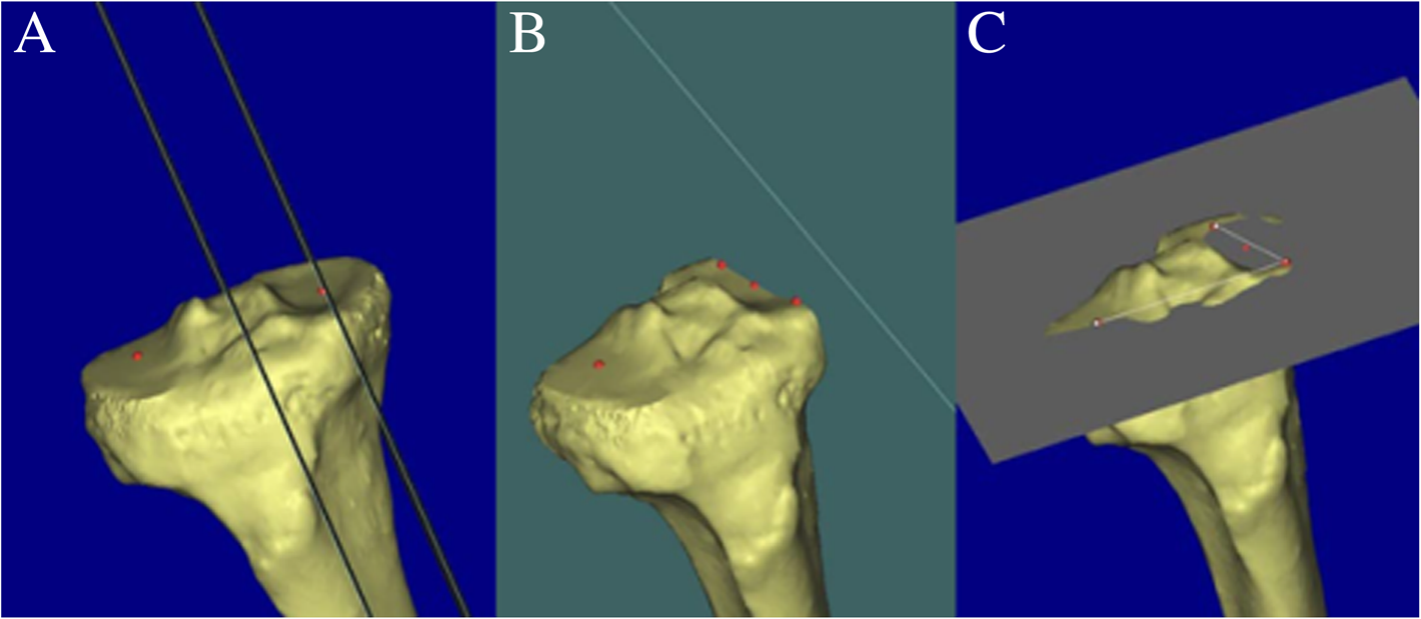
Planning steps for the plane of the TP. The line passing through a dwell point of the medial femoral condyle on a medial TP surface was drawn parallel to the tibial axis (**a**). Two points were plotted on the anterior and posterior rims of the medial tibial articular surface, defined within the sagittal plane coinciding with the dwell point of the femur on the medial TP (**b**). These two points and a dwell point of the femur on the lateral TP plane determined the plane of the TP (**c**)

The 3D relative alignments between the tibial axis, tibia plateau (TP), and tibial GP were determined within the anatomical coordinate system. The plane of the TP was determined by the following three points: a dwell point (defined as the most distal point of femoral condyle contact on the tibial surface with the knee in extension) of the femur on the lateral tibial articular surface, and two points on the anterior and posterior rims of the medial tibial articular surface defined within the sagittal plane that coincided with the dwell point of the femur on the medial tibia (Fig. 2).

**Fig. 3:**
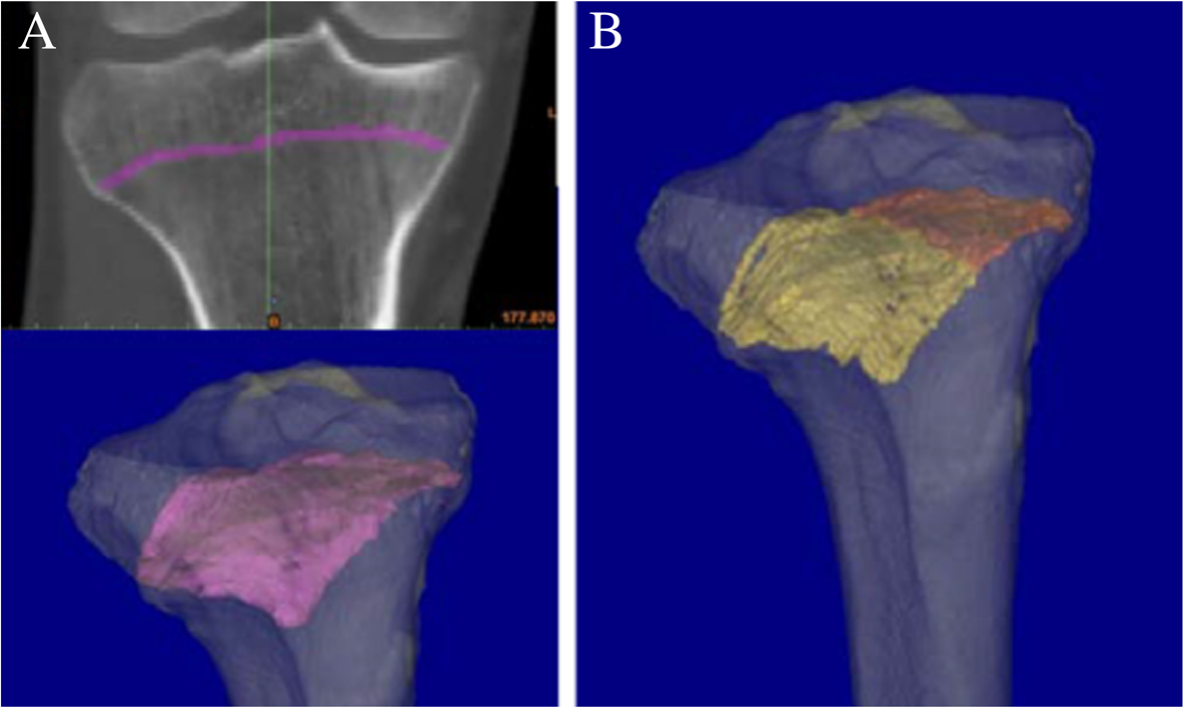
The 3D model of the GP. Shape extractions and 3D modeling of the tibial GP. The shape of the tibial GP was extracted and expressed as a 3D model using the livewire function and mask-editing tools of the Mimics® software (**a**). The 3D model of the GP was then divided into medial and lateral halves by the mid-sagittal plane (**b**)

**Fig. 4:**
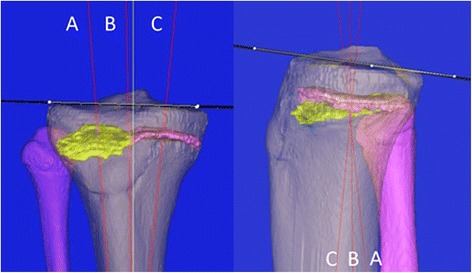
Orientation of the medial and lateral halves of the GP. Orientations of the GP were determined for the lateral half (A), whole (B), and medial half (C)

The geometry of the tibial GP was extracted using livewire function and mask-editing tools of the Mimics® software. The 3D model of the GP was then divided in medial and lateral halves about the mid-sagittal plane (Fig. 3). To determine the 3D orientation of the GP plane, moment-of-inertia axes were calculated for the 3D models of GP parts. A superior-inferior portion of the inertia axes were used for orientation of the whole GP plane. The orientations for medial and lateral half of the GP were also determined (Fig. 4). Measurement of each angle relative to TP was repeated three times to determine intra-observer variation analysis and calculate mean values. Intra-class correlation coefficients were 0.939, 0.903, and 0.903 for whole GP, GP lateral half, and GP medial half, respectively. All subjects participating in this study provided informed consent prior to enrolment, and all study protocols were approved by our institutional review board (Keio University School of Medicine, ID #20110245).

### Statistical analysis

Variance of the mean angle relative to the TP was compared between the medial and lateral halves of the GP and determined using an F-test. Values of *P* < 0.05 were considered significant, signifying that the variance of data is not equivalent between two groups.

## Results

The plane of the TP, which represented the tibial joint line as a surgical reference, was positioned in 2.3 ± 1.8° of varus and 11.3 ± 3.8° of posterior inclination with respect to the anatomical tibial coordinates (Table 1). The GP plane was positioned in 1.1 ± 1.9° of valgus and 0.1 ± 3.1° of posterior inclination relative to the anatomical tibial coordinate. With respect to the TP plane, the GP plane was inclined in 3.4 ± 1.5° of valgus in the coronal plane (Table 2).

**Table 1:** Angular orientation of TP and GP with respect to the anatomical coordinates of the tibia

	Angle to tibial anatomical coordinate [°]	
	Varus/valgus*	Posterior/anterior**
TP plane	2.3 (1.8)^#^	11.3 (3.8)
GP plane	− 1.1 (1.9)	1.1 (3.1)

*Positive value indicates varus inclination with respect to tibial anatomical coordinate

**Positive value indicates posterior inclination with respect to tibial anatomical coordinate

^#^Values are expressed as the mean (standard deviation)

**Table 2:** Angular orientation of GP with respect to TP

	Angle relative to the TP [°]	
	Varus/valgus*	Posterior/anterior**
Whole GP	− 3.4 (1.5)^#^	− 11.4 (3.1)
GP medial half	4.9 (2.9)	− 1.8 (2.5)^†^
GP lateral half	− 10.4 (2.4)	− 18.6 (4.0)

*Positive value indicates varus inclination with respect to the TP plane

**Positive value indicates posterior inclination with respect to the TP plane

^#^Values are expressed as mean (standard deviation)

^†^*P* = 0.015, variance of the mean angle relative to TP was not equivalent between GP medial half and GP lateral half

The shape of the GP plane differed between medial and lateral halves. The medial half of the GP was in 4.9 ± 2.9° of varus and 1.8 ± 2.5° of anterior inclination, and the lateral half was in 10.4 ± 2.4° of valgus and 18.6 ± 4.0° of anterior inclination relative to the TP. The posterior inclination of the medial half of the GP tended to follow that of the TP, while the posterior inclination of the lateral half did not, so that the GP plane apparently twisted about the center of the tibia.

## Discussion

The key finding of this study was that the GP plane was inclined in 3.4 ± 1.5° of valgus on average in the coronal plane relative to the TP plane, and the original TP line can be reproduced in reference to measured GP orientation on 3D CT patient data, providing reasonable guidance for the target bone resection angle of the tibia in KA- or AA-TKA. The lateral half of the GP plane appeared to demonstrate a relatively more reliable indicator of the TP line than the medial half of the GP for smaller standard deviation. The lateral half of the GP can be useful in patients with severe OA in which visualization of the medial GP is compromised.

Some recent studies have advocated a paradigm shift in the surgical principle of the TKA, from mechanical alignment to anatomical alignment, in expectation of the beneficial effects of replicating the natural slope of the tibial joint surface. In their discussions, detailed understanding of the proximal tibial anatomy, especially for the joint line orientation in both normal and arthritic knees, supported the concept of anatomical alignment [9, 10]. Limb alignment is highly affected by the geometry of both long bones and the femorotibial articular surface line (i.e., joint line orientation), which has been reported as variable among patients [11, 12] and would change with progression of OA and subsequent bone remodeling. Prediction and quantification of joint line orientation are difficult to achieve due to a lack of reliable bony landmarks that can be less affected by the arthritic change and deformities in the proximal tibia. Akhmedov et al. [13] reported that plane radiographic measurements of epiphysis-metaphysis angles did not show adequate reliability for the pediatric population. The present study found asymmetrical morphological characteristics of the GP plane and assumed that this resulted in variability in 2D radiographic analyses, causing difficulty of 2D measurement. In our method, determination of the GP plane from volumetric models can eliminate such measurement difficulty and may provide robust determination of the GP axis alignment with respect to the original TP articular line.

In our preliminary investigations on OA knees currently in progress, we encountered some cases with severe varus deformity in which proximal tibia vara was present. In some of those cases, the medial half of the GP plane was not available for clear visualization, while the lateral half of the GP plane was almost intact and available for 3D modeling to complete analysis, suggesting the usefulness of the lateral GP line as a reference for reproducing the original TP line in the coronal plane, even for knees with severe varus deformity.

The effect of joint line orientation on tibiofemoral load-sharing has remained poorly understood, apart from the impact of long leg alignment on implant survivorship. Victor et al. [14] reported that, based on long-leg weight-bearing radiographs, patients with pre-arthritic constitutional varus showed a joint line oriented parallel to the floor. Hutt et al. [15] proposed a similar effect on the natural joint line after KA-TKA. They suggested that less than 3° of varus joint line orientation relative to the floor may not increase the risk of premature implant loosening. Recent clinical trials of KA-TKA or AA-TKA have not suggested increased failure rates at 2 years of follow-up. The long-term survivorship of the modified implant alignment has yet to be investigated.

We acknowledge the following limitations of this study. First, the relatively small sample size and bias inherent in sample selection might have influenced the accuracy of the results. Most CT data were obtained from patients who had sustained rupture of the anterior cruciate ligament (ACL), in which tibiofemoral bone morphology played some role in ACL rupture. Second, lateral compartment OA was not considered an indication for this method, because the lateral half of the GP, which is more reliable than the medial half, is compromised and not available during 3D planning. Third, the purpose of this study was to establish a method for reproducing joint line orientation for possible application in predicting the pre-OA natural joint line, but the results shown here were all derived from non-OA knees. Analysis of OA knees is our ongoing project, and results from non-OA knees should be compared with those from OA knees in order to prove the feasibility of the GP plane concept in replicating the natural joint line.

## Conclusion

The present study indicated that the angular orientation of the original TP plane can be predicted with reference to the GP plane and provided a method for predicting joint line orientation using 3D CT data from non-OA knees. The GP plane was inclined in 3.4 ± 1.5° of valgus on average in the coronal plane relative to the TP plane. This method may be used as reasonable guidance for target bone resection angle of the tibia in KA-TKA.

## Abbreviations

3D: Three-dimensional
AA-TKA: Anatomically aligned TKA
ACL: Anterior cruciate ligament
CT: Computed tomography
GP: Proximal growth plate
KA-TKA: Kinematically aligned TKA
OA: Osteoarthritis
PCL: Posterior cruciate ligament
TKA: Total knee arthroplasty
TP: Tibial plateau
